# Illuminating austerity: Lighting poverty as an agent and signifier of the Greek crisis

**DOI:** 10.1177/0969776417720250

**Published:** 2017-07-23

**Authors:** Saska Petrova

**Affiliations:** The University of Manchester, UK

**Keywords:** Austerity, economic crisis, energy poverty, energy services, fuel poverty, Greece, illumination

## Abstract

Light – whether natural or artificial – plays multiple roles in the home: both as a material enabler of everyday life and as a device for exercising a variety of social relations. The post-2008 Greek economic crisis has endangered those roles by limiting people’s ability to access or afford adequate energy services. This paper focuses on the enforced lack of illumination in the home, and the strategies and tactics undertaken by households to overcome this challenge. I connect illumination practices and discourses to the implementation of austerity, by arguing that the threat of darkness has become a tool for compelling vulnerable groups to pay their electricity bills. The evidence presented in the paper is based on two sets of interviews with 25 households (including a total of 55 adult members) living in and around Thessaloniki – Greece’s second largest city, and one that has suffered severe economic consequences as a result of the crisis. I have established that the under-consumption of light is one of the most pronounced expressions of energy poverty, and as such endangers the ability to participate in the customs that define membership of society. But the emergence of activist-led amateur electricians and the symbolic and material mobilization of light for political purposes have also created multiple opportunities for resistance.

## Introduction

The post-2008 Greek debt crisis and associated austerity regime have received significant academic attention. There is now considerable knowledge on the political economies that drove the events of the last 8 years, from the systemic roots of the sovereign debt crisis, to its transformation into a full-blown recession at an unprecedented scale (Featherstone, 2015; Katsimi and Moutos, 2010; Mavroudeas, 2014). A distinct body of scholarship has examined the multiple economic and spatial displacements that have affected Greek society, often involving the augmentation of existing inequalities (Gialis and Leontidou, 2014; Matsaganis and Leventi, 2013). Political contestations of the austerity regime have also been scrutinized in this context (Dalakoglou and Kallianos, 2014; Douzinas, 2013; Mylonas, 2014), highlighting the multiple articulations of resistance via spontaneous and organized formations. Yet there has been comparatively less research into the everyday micro-scale experiences of the crisis, as well as the ways in which austerity policies have become implicated in shaping people’s practices and identities (Georgakopoulou, 2014; Kaika, 2012; Knight, 2013; Rakopoulos, 2014).

The Greek economic crisis and the subsequent imposition of an austerity regime (Alejos and Paz, 2013; Peck, 2012) have led to increased end user prices and taxation, while driving down incomes. Social safety nets have been dismantled, pushing large parts of the population into hardship. One of the consequences of this set of circumstances has been the rise of energy poverty (Bouzarovski and Petrova, 2015): a condition underpinned by the lack of socially- and materially-necessitated domestic ‘energy services’ (Modi et al., 2005), otherwise understood as the benefits that energy brings to household well being – space cooling, lighting, heating, and so on. Energy poverty is a form of material deprivation, and as such extends beyond household incomes and utility prices to encompass the state of the housing stock – particularly the energy efficiency of the built fabric, heating systems and appliances – as well as the wider institutional, spatial and cultural underpinnings of infrastructural service provision (Bickerstaff et al., 2013). Traditionally well-known and widely discussed in the UK and Ireland under the aegis of the ‘fuel poverty’ debate (Boardman, 2010; Jansz and Guertler, 2012), this phenomenon has recently entered the lexicon of decision-makers and scientists across a number of European countries, including Greece (Bouzarovski et al., 2015; Thomson et al., 2016). But even within the Greek context, the debate has primarily been preoccupied with issues of space heating (and less so, cooling), with other energy services receiving comparatively little or no attention. What is more, relatively little is known about the manner in which neoliberal economic policies interact with household-level strategies to produce new vulnerabilities at the nexus of imagined and material infrastructural landscapes (Becker et al., 2016; Bouzarovski et al., 2015; Luque-Ayala and Silver, 2016).

In light of such knowledge gaps, this paper explores the relationship between discourses and practices of austerity in Greece, on the one hand, and experiences of energy poverty, on the other. The latter is scrutinized via the inability to access or afford adequate levels of illumination in the home. I examine the manner in which austerity both leads to and is predicated upon the economically and materially enforced lack of light in people’s homes. Within this overarching purpose, the paper aims to: (i) uncover the circumstances under which the under-consumption of lighting leads to forms of socio-technical deprivation; (ii) investigate how the everyday links between lighting and poverty are articulated via the material, technical and economic underpinnings of home; and (iii) interrogate the literal and figurative use of the ‘fear of darkness’ (Vannini and Taggart, 2013) in promoting the austerity agenda. One of the starting premises of the paper is that needs and practices surrounding energy have been used as a tool for the construction of crisis, particularly in terms of compelling vulnerable groups to pay their electricity bills. This has led me to argue that driving forces of lighting deprivation are energopolitically-embedded (Boyer, 2011; Szeman, 2014) via institutional and symbolic devices aimed at regulating and disciplining the infrastructural conduct of the Greek people. I also use insights from the ‘anthropology of luminosity’ (Bille and Sørensen, 2007) to underline how people’s lived experience and multi-scalar practices of power are implicated in the creation of multiple ‘lightscapes’ of crisis.

The evidence presented in the paper is based on home-based interviews with 25 households (including a total of 55 adult members) living in and around Thessaloniki – Greece’s second largest city, and one that has suffered severe economic consequences as a result of the crisis (Slini et al., 2015). The households were interviewed on two occasions – during the summer of 2013 and the winter of 2014 – with the aim of exploring the different types of challenges that they may have faced during the heating and cooling seasons (Thessaloniki’s climatic conditions are such that both are markedly present (Slini et al., 2015)). The interviewees were identified based on informal contacts as well as acquired knowledge, with each interview lasting between 1 and 2 hours. All of the interviews were undertaken in Greek and tape-recorded, after which they were transcribed and translated into English. In cases where they have been cited this paper, however, the interviewees’ names have been changed for ethical purposes. The interviews were analysed inductively and interpretively, in combination with an extensive corpus of documentary data – news items, think tank reports, policy statements and legal acts. A significant additional contribution to the evidence base was made by 14 ‘expert’ interviews with local and national decision-makers, political party representatives, community activists and publicly engaged academics, undertaken between 2013 and 2016. This was supplemented by an analysis of quantitative energy data sourced from the Hellenic Statistical Authority and the Greek Energy Observatory.

Other than the introduction and conclusions, the paper has four sections. The first of these offers a general overview of the fragmented body of scholarship on ‘light poverty’ across the world, as well as the emergent literature on the political and cultural construction of light as a place-making agent. This is followed by a review of austerity-induced patterns of inadequately-lit homes in Greece, after which I focus on the multiple domestic meanings of this energy service, and the inability of households to secure it to an adequate level. The ensuing section underlines how discourses of light and darkness have been implicated in attempts to advance particular types of energy policy and regulation under the aegis of the austerity regime. I also discuss the political utilization and potential of light-related energy services as an emancipatory project to challenge neoliberally-inspired structural adjustment policies.

## Deconstructing domestic light and darkness

2015 was the ‘International Year of Light’ – a global United Nations-led initiative with the declared aim of ‘raising global awareness about how light-based technologies promote sustainable development and provide solutions to global challenges in energy, education, agriculture and health’ (UNESCO, 2015). The framing of this programme reflects some of the mainstream policy concerns in the domain of ‘light poverty’, which have been preoccupied with providing technological solutions to address the lack of ‘clean light’ for the ‘1.3 billion people living without electricity worldwide’ (Vozzi and Ramponi, 2016: 2). ‘Clean light’ in such contexts has mainly signified electric light, because the lack of access to this energy carrier has meant that people are forced to use candles or kerosene lamps, which are known to have serious effects on health and well-being (Lam et al., 2012). The inability to provide adequate artificial light has thus been seen as a failure of modernity, with urban blackouts acting as indicators of vulnerability of cities to infrastructural collapses (Gandy, 2017).

Traditionally, however, ‘light poverty’ has been bound up with development-orientated electrification efforts, focused on increasing economic output and expanding grid coverage via large-scale infrastructural approaches and the provision of financial capital via neoliberal frameworks (Barnes, 2007; Xu, 2006). The inability of such approaches to deliver meaningful reductions in the global number of people who lack access to ‘modern energy’ (Bazilian et al., 2010) has contributed to a shift in focus towards ‘the productive uses of energy’ and a recognition of ‘the tremendous impact that energy services have on education, health, and gender equality’ (Cabraal et al., 2005: 117). There has been a growing emphasis on off-grid solutions such as small scale solar lighting and sub-centralized LED lamps (Hong and Abe, 2012; Yaqoot et al., 2014) even if there is evidence to suggest that ‘the development implications of solar electrification are closely linked to its role in enabling the use of “connective” devices’ (Jacobson, 2007: 144) such as televisions, radios and mobile phones. At the same time, it has been highlighted that the take-up of solar technologies has been bound up with issues of ‘financial exclusion, weak governance, and passive NGO and customer participation’ (Wong, 2012: 110) in addition to a much wider set of cultural meanings and practices of power (Winther, 2013).

The nature and content of the debate change radically when the emphasis is placed on developed-world countries, including those in Europe. They do not recognize ‘light poverty’ as a distinct issue, even if this type of energy service has often been mentioned in the context of wider difficulties with fuel or energy poverty. Official definitions of fuel poverty in the UK incorporate a variety of final energy forms, thus acknowledging the broader finding that ‘lighting and heating dominate the energy use of fuel-poor households’ (Roberts, 2008: 4472). But Simcock et al. (2016) have found that policy and advocacy discourses are disproportionately focused on heating, with only one NGO definition of fuel poverty – by the National Right to Fuel Campaign – vaguely mentioning lighting as a distinct energy service in the context of fuel poverty.

It should be mentioned that lighting is an important subject in the distinct literature on the rebound effects of energy efficiency interventions in the home (Chitnis et al., 2013). The basic understanding of the ‘rebound effect’ is based on the argument made by Jevons (1865) who suggested that more efficient industries do not necessarily lead to lower energy consumption because people may produce and consume more goods as a result of lower unit costs. There is also now a significant body of work on the rebound effect in the case of residential lighting (Greening et al., 2000; Lv et al., 2016; Mahapatra et al., 2009; Mills and Schleich, 2014; Saunders and Tsao, 2012; Schleich et al., 2014). Still, such work primarily operates with quantitative approaches, and rarely relates to energy poverty scholarship. Within the European context, one of the very few insights into energy poverty-related light deprivation is provided by Brunner et al. (2012), who highlight the different practices of reduced illumination adopted by households, from reducing the number of light sources or lit rooms, to the reliance on indirect lighting from appliances such as televisions. In line with much of the literature on the ‘social loading’ of energy consumption (e.g. Wilhite and Lutzenhiser, 1999), Brunner et al. found that ‘even if many of the people interviewed refer to more or less developed habits of reducing the lighting in their everyday illumination practices, there are many examples where a reduction is considered inadequate and full illumination of the house is opted for… particularly in the presence of visiting friends or relatives’ (Brunner et al. (2012: 56).

The disconnect between developing and developed-world literatures on energy poverty-induced light deprivation points to the need for moving towards a common conceptual framework and vocabulary to understand this problem. Regardless of whether the deficiency of domestic lighting services is caused by inadequate infrastructural access or the poor affordability of energy, its outcomes are the same: households end up living in an insufficiently-lit home, which prevents them from participating in the patterns, customs and activities that define membership in society (Townsend, 1979). This suggests that ‘light poverty’ is primarily a socio-political construct, embedded in the norms, meanings and expectations associated with the technologies and levels of infrastructural service provision that can satisfy household energy needs (Day et al., 2016; Shove and Walker, 2014).

Of relevance here is the emergent body of thought on the ‘cultures of lights’ (Kumar, 2015), which has argued, inter alia, that the practice of illumination lies at the fulcrum of material and affective dimensions. Thus, the quality and quantity of light in the home ‘indicate the material possessions of the household’ and it ‘has a critical role in establishing and reinforcing honour (Kumar, 2015: 67). As Kumar explains, ‘having a light in front of the house, in the space that is publically visible’ signifies the status of the household and it ‘upholds their honour in the society’ (Kumar, 2015: 61). In this case Kumar links the notion of honour with care and ‘the material capabilities for taking care of the guests well’ (Kumar, 2015: 64). It has also been shown that the interactions between light, darkness and space are experienced and perceived differently depending on age, social class and gender (Day, 1999; Edensor and Millington, 2013), with the ‘micro-features of lighting’ (Pain et al., 2006: 2068) playing a key role in people’s experiences of place.

The significant social science literature on blackouts (e.g. Bennett, 2005; Nye, 2010; Trentmann, 2009) also deserves mention – it has highlighted the diverse infrastructural, institutional and material implications of power cuts for the conduct of everyday life and the regulation of the economy more generally. But this body of work has rarely communicated with contributions on the meaning and practice of ‘mundane’ illumination in the indoor environment of the home, which in turn remains disconnected from energy poverty scholarship. It is in the midst of these multiple conceptual lacunae that I locate the contribution of my study. Echoing Shaw’s call ‘to establish whether the darkness that remains in the face of expanding capitalism is a darkness that leaves certain people behind’ (Shaw, 2015: 641), I seek to understand how the constructions of austerity and crisis are bound with both the experience and threat of light deprivation – from the micro-spaces of the home to the nationwide spectre of blackouts.

## Dark homes and energy poverty in Greece

Greece is among the small number of European countries (outside the British Isles) where energy poverty has received significant scientific and policy attention. There is a substantial body of English-language scholarship on the systemic embeddedness of energy consumption inequalities and inequities in this country. Donatos and Mergos (1989) for example, have examined the impact of the 1973/1974 and 1978/1979 oil crises, highlighting that the overall per capita energy use in Greece during the 1970s and 1980s increased at a faster rate compared to other member states of the International Energy Agency. At the same time, the crises showed that the demand for liquid fuels is income elastic – thus foreshadowing some of the problems that emerged in recent years (Donatos and Mergos, 1989). Historical trends of rising energy demand, however, have not been accompanied by the development of adequate socio-technical systems of provision; beyond its recent introduction to inner-city Athens and a few urban centres on the mainland, piped natural gas is not available in most of the country. It has been statistically demonstrated that energy consumption in Greece – and economic growth more generally – is closely related to infrastructural availability (Azam et al., 2016).

As was noted above, the post-2008 crisis has brought about energy tariff rises – accompanied by tax increases involving extra levies being added on top of electricity bills – as well as a rapid drop in income and the scaling back of state-sponsored social protection. It is worth mentioning that electricity bills in Greece are issued every two months based on an estimate of previous consumption, which is metered every fourth month. At the end of the fourth month, consumers pay a bill that covers any differences in real consumption. People find the payment of the four-month bill difficult – as it can be several times higher than the usual bill – especially after a long summer with high temperatures, or a cold winter. In combination with Greece’s poor quality and energy-inefficient housing stock, this set of conditions provides fertile ground for the expansion of energy poverty (Healy, 2004). Several comprehensive programmes of research have pointed to the extensive consequences of the economic crisis on human welfare and well-being in energy terms. For example, a field survey undertaken in three northern Greek cities (including Thessaloniki) found that household energy consumption for heating was reduced by approximately 60% between 2008 and 2012 – principally due to a drop in household oil and gas demand – while domestic electricity consumption remained relatively stable due to the use of this carrier for providing thermal comfort via air conditioning devices. But retail sales of wood and pellets increased by almost 720% since the beginning of the recession (Slini et al., 2015). It also transpired that poorer households lived in less energy-efficient dwellings, with 2% and 14% of high- and low-income households, respectively, being defined as energy poor by the authors (Slini et al., 2015). Research with 50 households during the winter 2012–2013 showed that ‘indoor temperatures are much below the accepted standards and in many cases place in risk the health and even the life of the residents’, with ‘a high fraction of households’ not using heating energy at all (Santamouris et al., 2014: 61).

All of the work reviewed above, however, rarely refers to energy practices beyond heating, and the wider political and institutional contexts through which domestic energy deprivation is produced and conditioned. Dagoumas and Kitsios’ (2014) study is one of the few to link household-level energy poverty patterns with the socio-technical issues faced by utility companies. In addition to highlighting the considerable effects of the economic crisis ‘on the electricity consumption and on the capability of people to pay their bills’ (Dagoumas and Kitsios, 2014: 267), it also emphasizes that non-payment on a massive scale can create ‘serious liquidity problems for the [Greek] Public Power Corporation [PPC]’ thus potentially transforming an energy poverty issue to an energy security issue (Dagoumas and Kitsios, 2014). In order to address increasing debt, PPC announced the opportunity to use prepayment meters at the beginning of 2017. Still, the use of such devices is not common in Greece. Kolokotsa and Santamouris (2015) have, notably, considered the issue of ‘visual discomfort’ in their review of the indoor environmental quality and energy consumption studies for low income households in Europe. Even if their study is focused on ‘natural light’ rather than energy-poverty related deprivation from artificial lighting, they do underline the importance of light for productivity, stress reduction and health. Citing Rybkowska and Schneider (2011) they emphasize that ‘dark homes’ are a pervasive problems across Europe, including Greece – where they affected up to 7% of the population in 2012. Other than this statistic, the only other nationally available figure on lighting comes from the Hellenic Statistical Authority, which reported that the average Greek household spent **€**35 on lighting in 2012 – not an insignificant figure given decreasing family budgets.

## Practicing the crisis: Lighting deprivation as a cultural signifier

The austerity-induced plight of energy poor households in Greece has received extensive attention in the local and international polity. Greek newspapers widely cited a *Handelsblatt* article titled ‘When the lights go out in Greece’, evoking the incident in which a 13-year-old girl died of fumes in her own house, which had previously been disconnected due to unpaid bills. Having emphasized the 60% increase in energy bills since 2007, the article provides extensive information about the ‘illegal’ reconnections of power and related initiatives, while highlighting the economic losses suffered by the Public Power Corporation (PPC) from unpaid bills. It stresses a governmental initiative aimed at creating committees to ensure that the disconnections are not made in the case of ‘vulnerable’ families (Life.gr, 2012). Similarly, a *Vice* magazine report that received extensive attention in the Greek social media sphere used images of a family living in darkness to illustrate the lack of access to energy infrastructure in an inner-city Piraeus neighbourhood. The story describes how the children belonging to this family ‘go to night school so they can study through daylight’ (Karavaltsiou, 2014). The lack of electricity means that they have to use candles at night, creating a fire hazard that requires constant vigilance.

Among the interviewed families in Thessaloniki, lighting – and associated issues of deprivation – was one of the key themes that emerged from the inductive analysis of household interviews. Lighting was discussed in varied ways embedded in notions of self-deprivation, control and gender as well as identity and stigma. Most of the households pointed out the need to control or reduce the use of specific domestic energy services, with lighting-related tactics being among the first ones to be practised in this context. For the household of 32-year-old ‘Stefania’, and 34-year-old ‘Nikos’ – working as a teacher and bus driver, respectively, while parenting a young child – ‘the lights are not on without a reason nor is the TV’ because doing otherwise would have increased their financial outlays. This was despite the fact that they lived in their own apartment and were in the process of building a house.

Even households with higher incomes, such as that of ‘Alexandros’ (a 36-year-old IT specialist), ‘Nikoleta’ (a 32-year-old human resources employee) and their 9-year-old daughter, made sure that the lights were off when they ‘did not need them’. Living in a private rental flat, they were worried about rising housing expenditure in the face of falling incomes as well as rising utility, transport and food prices. At the other end of the spectrum were families that had been struggling to make ends meet even before the crisis. Such was the case of a 62-year self-employed electrician (‘Themis’), who lived with his son ‘Alek’ and daughter ‘Thea’, both students in their early 20s. While this household’s low income meant that they were eligible to receive benefits from the state, they were nevertheless very thrifty with their energy use, in addition to closely measuring and regulating electricity consumption. They were especially concerned with ensuring that their electricity demand stayed persistently low:

Some people continue past practices, like leaving the lights on, using night lights or using outdoor lights. They do not understand that they can economize on the electricity bill by adopting better practices (Themis, winter 2014).

Undertaking such a complex array of energy-saving strategies and tactics required significant amounts of time and labour. This is where the highly gendered character of the Greek economic crisis (Karamessini, 2013; Vaiou, 2014) became particularly visible. Within almost all of the interviewed households, the traditional role of women as ‘homemakers’ meant that they had to take upon themselves the task of not only managing the family budget, but also dealing with the convoluted technicalities of juggling different energy uses while keeping bills low. Lighting was one of the most demanding challenges in this context, often creating conflicts among different household members that had to be resolved by women – in the form of micro-austerity practices that cut against the grain of women’s conventional roles as care-givers (Gonzalez et al., 2014). As pointed out by 44-year-old truck driver ‘Alekos’, who lived with his wife ‘Adonia’ and two small children in a rented apartment: ‘My wife pays for the electricity and controls when the lights are on or not’. In three of the interviewed households – all of which lived at the outskirts of the city – women’s key role in weaving together the networks of non-market and non-capitalist economic practices that sustain household well-being in times of crisis (Vaiou, 2016) was also apparent in the procurement of low-cost energy resources via informal channels.

Light’s material and social agency in the production of domestic space – particularly in terms of generating comfort and ambience – meant that reductions to illumination had significant impacts not only on a household’s well-being but also its functioning and standing among friends and relatives. Energy poverty thus allowed austerity to enter the intimate aspects of everyday life, exercised via private spaces of the home. For ‘Dosia’ – a 38-year-old secretary in the private sector – making sure that the electricity was never cut off was a primary concern, even if this meant radical cuts to her household’s energy consumption. She was worried about how her ability to light the house adequately would be perceived by visitors to her house and the local community more widely.

Such aspects were also of importance to households that used the home as a source of economic activity, especially in cases where this involved interactions with outside parties. Forty-seven-year-old ‘Alexandra’s’ bed-and-breakfast business was under threat by her financial ability to pay energy bills. She was ‘scared of power cuts’ because her business would be ‘ruined without hot water and lighting’. Similar statements were provided by individuals who conducted informal activities – cooking, sewing, weaving and knitting, and caring – in the domestic environment. Thus, and to summarize, the economic crisis both foregrounded and amplified light’s multiple domestic functions and meanings, as well as the diverse economic activities that households are capable of articulating in relation to this service.

## Constructing and contesting the crisis

Moving beyond household adaptations to the crisis – and associated inventions, challenges and frustrations – energy poverty generated a feeling of anger and revolt among many of my interviewees. This was particularly pronounced in relation to the government’s decision to incorporate a range of non-energy taxes and levies in electricity bills (especially the notorious ‘*haratsi*’ – a universal tax on all forms of property ownership, established in 2011). The state’s use of a backdoor mechanism to embed austerity policies in the provision of an essential residential service was perceived as highly unjust and unfair. With new taxes being clearly displayed on power bills, however, the surveyed households were acutely aware of the burden that austerity placed on their everyday life in this manner. During the winter interview, Alekos’ wife Adonia showed us her most recent power bill: only 40% of the charges incurred accounted for electricity consumption, with the remainder covering various taxes and levies.

Many interviewees were worried that additional taxation would endanger their ability to pay for energy services: 35-year-old ‘Nikoleta’ – a pharmacist who lived with her self-employed husband and two children in a privately-owned house – listed ‘losing one’s job’ and ‘future tax increases’ as the two key factors that might lead to her household’s electricity being cut off. At the same time, ‘Olga’ – 41, unemployed, living in a rented house with young twins and her 42-year old husband Maximos, otherwise a policeman – thought that:

…the additional costs on the electricity bill should not be there… it is crazy that I have to pay all these additional taxes to the PPC. They are not responsible. We should pay the PPC only for the things that the PPC provides us (Olga, winter 2014).

Similar concerns were expressed by the household of ‘Evania’ (53, self-employed in the private sector), ‘Kristos’ (54, self-employed in the private sector), ‘Lazaros’ (self-employed in the private sector), and ‘Isidora’ (29, a part-time public sector employee). Consisting of a couple with adult children who mainly derived their income from a family-owned tourist agency, this family emphasized that ‘the increase in electricity prices combined with rising taxation is what puts a strain on living costs’, while ‘income falls and expenses increase’. They thus worried about reaching a point where they will be unable to pay their bills, ending up in a state of disconnection and darkness. This anxiety was not only expressed at the household scale, however – cuts to local authority budgets led to dramatic decreases in street lighting:

In the evenings, many municipalities turn into… dark communities and, as a result, criminal activities are increasing, and, as a consequence of this, the feeling of insecurity among citizens also rises (GR Reporter, 2013).

As was noted above, the spectre of darkness was also present within national-level discourses, in the form of frequent media reports and political threats about the financial solvency of Greek power utilities as a result of bill non-payment and arrears. In 2012, it was reported that ‘Greece faces a widespread danger of blackouts, as the economic crisis leaves PPC without cash to pay for gas imports and operation of power stations’ (newsnow.gr, 2012). Even if this claim was denied by PPC officials, the same article cited a number of energy experts who emphasized that ‘a blackout is certainly a possibility’ (newsnow.gr, 2012). Thus, light provided a metaphorical and material device to domesticate the infrastructural implications of the economic crisis, while disciplining people into implementing the austerity programme at the scale of everyday life. But my interviews also revealed an ambiguity to the material darkness stemming from energy poverty. A number of households emphasized that reductions in domestic lighting, paradoxically, made austerity more visible and prescient, while increasing the closeness and intimacy of household members in new and unexpected ways. At the same time, mass darkness provided a form of symbolic political protest at a rally against austerity in the city of Patras, where the local mayor stated that:

Tonight Patras is switching its lights off to show our leaders and their foreign overlords what it means to leave the world in darkness. We switch off the lights of the city, but we turn on the lights of the struggle against the *haratsi*. None of our fellow citizens must be left and will not be left without power. We will devote our common struggle to this (Newsit.gr, 2011).

The anti-austerity movement also utilized the practice of reconnection – and the trope of bringing back light to the people – as a form of active political resistance. Instances of political activists who made physical interventions to the grid so as to provide electric power for disconnected households became widely publicized in the Greek and international media:

We are a movement of political defiance. We restore electricity in 15 houses per day. We help people suffering from poverty (euronews.com, 2013).

An interview with a representative of the Antarsya movement – also known as the Front of the Greek Anticapitalist Left – revealed that a systematic strategy was developed in this context:

We had a three-pronged approach. One option was legal aid; we would send a non-judicial notice of default to PPC, in order to stop the electricity cuts because of the *haratsi*. People would pay for the electricity consumed, but they would not pay the *haratsi*. Not paying a form of tax like *haratsi* does not justify cutting off electricity. The second option was to intervene in disputes by going to the office of the local PPC branch director and demand that vulnerable households are reconnected while making an arrangement for their electricity bills to be sorted out…. The third, and in my opinion, the most important activity, was the demonstration of solidarity in practice, by forming teams of people who had the knowledge and the skills to reconnect electricity where it was cut off. So what we did was put posters around the streets calling people to attend meetings, and at the same time we informed them that if they had issues with electricity cuts by PPC they could call the team and they would reconnect it for them (personal communication, political activist, January 2014).

The activist stated that the PPC faced major problems in instances where they tried to enter people’s homes in order to take meter readings or implement disconnections. In a further demonstration of the entrance of neoliberalism in the governance of the energy sector, it was reported that such tasks had been passed onto private subcontractors. Nevertheless, electricity disconnections generated a wider political dialogue in Greek society, as highlighted by the Συνασπισμός Ριζοσπαστικής Αριστεράς, or the Coalition of the Radical Left (SYRIZA) political party’s energy department co-ordinator:

We are working on different levels: in the parliament; having an open dialogue with different actors in the energy sector; reaching out towards society by publicizing our ideas (personal communication, political activist, June 2014).

## Conclusions

I have sought to scrutinize how the materially, economically and politically enforced lack of lighting in the home is embedded in the ‘appliances, infrastructures, social norms and human action’ (Bates et al., 2012: 108) associated with the post-2008 Greek austerity regime. In relation to the first aim of the paper outlined in the Introduction above, I found that under-consumption of light is one of the most pronounced expressions of energy poverty, and as such was common among the households that I interviewed and, by implication due to secondary evidence, among the Greek population more widely. ‘Light poverty’ was expressed in different ways – from qualitative and quantitative reductions in illumination, to the substitution of more modern carriers, primarily electricity, with less technologically complex sources of energy at the lower rungs of the ‘energy ladder’ (Sovacool, 2011). While the physical health implications of this form of material deprivation are relatively poorly researched, it became clear that ‘light poverty’ is deeply entangled with social identity and status. This brings me to the second aim on the paper – on the everyday links between lighting and poverty in the domestic environment – where it transpired that socio-technical infrastructures of the domestic environment both shape and are shaped by this energy service. The lack of illumination affects household functionings in terms of, inter alia, family relations, gender identities, the provision of hospitality and the articulation of alternative economic practices. Lighting is also a key instrument of austerity. In relation to the third aim of the paper, I uncovered that needs and practices surrounding illumination are used as a tool for the construction of crisis and controlling vulnerable groups. But the emergence of activist-led amateur electricians and the symbolic and material mobilization of light for political purposes have also created multiple opportunities for resistance and resilience.

The lightscapes of crisis uncovered by my research are saddled with internal contradictions. Reflecting Kumar’s (2015) findings, they are both material and discursive – affecting everyday life while being omnipresent in public discourses. They are an integral part of the neoliberal regulation of austerity even if they involve the articulation of different forms of non-market and non-capitalist practices. And, perhaps most importantly, they embed a form of state and corporate control at the same time as offering fertile possibilities for emancipation. The complex relationships between crisis and lighting poverty calls for rethinking the notion of lightscapes (Bille and Sørensen, 2007) itself, because it has transpired that light can be harnessed as a political agent at a multiplicity of scales in addition to being an active component of the social life of human communities. The suggestion that ‘light has been influential as a metaphor of existence, clarity and truth’ (Bille and Sørensen, 2007: 272) is instructive here, as it becomes clear that the threat of darkness, on the one hand, and the possibility or moment of illumination, on the other, actively produce spaces of power and politics. In a sense, austerity allows for domesticating the fear of darkness, which is transferred – both physically and metaphorically – from the domain of public space into the insecurities and uncertainties that underpin energy circulations in the home.

Constraints on time and money meant that the research leading to this paper was limited to a relatively bounded set of places and people. But some the methods I used – particularly the tracing of people’s energy-related social practices over different periods of time – as well as the study’s emphasis on the everyday experience and articulation of the economic crisis, open up a series of broader questions about energy demand for lighting in the context of austerity regimes. In the first instance, this extends to the role of light services in the causes and consequences of material deprivation – it remains unclear how the highly socially contingent need for illumination in the home can be judged and weighed within the context of wider debates on distributional and procedural justice in the energy sector (Simcock et al., 2016). The relationship between artificial and natural light in this context deserves particular attention, given the almost complete absence of scholarship and data on inadequately-lit homes as a result of economic factors, as well as the enforced lack of access to modern energy carriers for lighting across the world. And last but not least, numerous questions remain to be asked with regard to light’s role in the domestication of austerity, both in terms of the representation, practices and experiences of disconnection, as well as the manner in which large-scale narratives of crisis connect to strategies at the household scale.
